# Comparative transcriptome analysis of *Arabidopsis thaliana *infested by diamond back moth (*Plutella xylostella*) larvae reveals signatures of stress response, secondary metabolism, and signalling

**DOI:** 10.1186/1471-2164-9-154

**Published:** 2008-04-09

**Authors:** Jürgen Ehlting, Sunita G Chowrira, Nathalie Mattheus, Dana S Aeschliman, Gen-Ichiro Arimura, Jörg Bohlmann

**Affiliations:** 1 Michael Smith Laboratories, University of British Columbia, 2185 East Mall Vancouver, B.C., V6T 1Z4, Canada; 2 Department of Botany, University of British Columbia, 6270 University Blvd Vancouver, B.C., V6T 1Z4, Canada; 3 Department of Forest Sciences, University of British Columbia, 2424 Main Mall Vancouver, B.C., V6T 1Z4, Canada; 4 Department of Statistics, University of British Columbia, 6356 Agricultural Road, Vancouver, B.C., V6T 1Z4, Canada; 5 Centre for Forest Biology & Department of Biology, University of Victoria, PO Box 3020 TN CSC, Victoria, B.C., V8W 3N5, Canada

## Abstract

**Background:**

Plants are exposed to attack from a large variety of herbivores. Feeding insects can induce substantial changes of the host plant transcriptome. *Arabidopsis thaliana *has been established as a relevant system for the discovery of genes associated with response to herbivory, including genes for specialized (i.e. secondary) metabolism as well as genes involved in plant-insect defence signalling.

**Results:**

Using a 70-mer oligonulceotide microarray covering 26,090 gene-specific elements, we monitored changes of the Arabidopsis leaf transcriptome in response to feeding by diamond back moth (DBM; *Plutella xylostella*) larvae. Analysis of samples from a time course of one hour to 24 hours following onset of DBM feeding revealed almost three thousand (2,881) array elements (including 2,671 genes with AGI annotations) that were differentially expressed (>2-fold; p[t-test] < 0.05) of which 1,686 also changed more than twofold in expression between at least two time points of the time course with p(ANOVA) < 0.05. While the majority of these transcripts were up-regulated within 8 h upon onset of insect feeding relative to untreated controls, cluster analysis identified several distinct temporal patterns of transcriptome changes. Many of the DBM-induced genes fall into ontology groups annotated as stress response, secondary metabolism and signalling. Among DBM-induced genes associated with plant signal molecules or phytohormones, genes associated with octadecanoid signalling were clearly overrepresented. We identified a substantial number of differentially expressed genes associated with signal transduction in response to DBM feeding, and we compared there expression profiles with those of previously reported transcriptome responses induced by other insect herbivores, specifically *Pieris rapae*, *Frankliniella occidentalis*, *Bemisia tabaci*,*Myzus persicae*, and *Brevicoryne brassicae*.

**Conclusion:**

Arabidopsis responds to feeding DBM larvae with a drastic reprogramming of the transcriptome, which has considerable overlap with the response induced by other insect herbivores. Based on a meta-analysis of microarray data we identified groups of transcription factors that are either affected by multiple forms of biotic or abiotic stress including DBM feeding or, alternatively, were responsive to DBM herbivory but not to most other forms of stress.

## Background

*Arabidopsis thaliana *has emerged as a useful system for genomic studies of plant insect-interactions [1-6]. Because of the large amount of genomic information available for Arabidopsis, it is possible to perform comparisons of gene expression profiles across many different conditions or treatments including various forms of interactions with herbivores and pathogens [1,2]. With regard to specific pathways involved in plant defence against insects, the Arabidopsis genomic resources have much advanced, for example, the discovery of genes and proteins of secondary metabolism (specifically glucosinolate, phenolic, and terpenoid metabolism) [7-11], as well as genes involved in plant-insect defence signalling [12-14].

Previous large-scale gene expression microarray analyses of Arabidopsis-herbivore interactions involved plants affected by *Pieris rapae *(larvae of cabbage white butterfly), *Spodoptera littoralis *(larvae of mediterranean brocade), *Frankliniella occidentalis *(western flower thrip), *Bemisia tabaci *(silverleaf whitefly nymphs), *Brevicoryne brassicae *(cabbage aphid), and *Myzus persicae *(green peach aphid) [1-5,15]. These insects represent leave-chewing larvae (*P. rapae *and *S. littoralis*) as well as cell-sucking (*F. occidentalis*) or phloem sap-feeding (*M. persicae*, *B. brassicae*, *B. tabaci*) adults with *P. rapae *and *B. brassicae *being specialist herbivores adapted to members of the *Brassicaceae *as their hosts. The present study complements previous work with an analysis of Arabidopsis rosette leaves fed upon by larvae of a different leave-chewing specialist herbivore, *Plutella xylostella *(diamond back moth – DBM). DBM larvae feed on several crucifer plants and are a frequent pest of agricultural crops including cabbage, broccoli, cauliflower, and rape [16].

Overall, our findings from a fully replicated time-course transcriptome analysis of Arabidopsis challenged by DBM larvae identified almost three thousand (2,881 array elements; 2,671 genes with AGI annotations) differentially expressed genes (>2-fold; p[t-test] < 0.05) and several distinct temporal patterns of changes of transcript abundance with prominent changes of transcripts associated with stress response, secondary metabolism, and signalling. In addition, we provide a first comprehensive meta-analysis of array data of herbivore-induced Arabidopsis transcription factors, which identified insect-induced transcription factors that are also affected by other forms of biotic or abiotic stress as well as transcription factors that appear to be more specific to the insect-induced response.

## Results

### Overall changes of the Arabidopsis leaf transcriptome in response to DBM

Arabidopsis rosette leaves (ecotype *Ler*) were challenged with feeding DBM larvae (third to fifth instars). For microarray gene expression profiling, rosette leaves were harvested after 1 h, 4 h, 8 h, and 24 h of continuous DBM feeding. For each time point leaves were also harvested from unchallenged control plants. In order to obtain enough plant material for RNA isolation (leave material became limiting in particular at the later time points of DBM feeding) each treatment and time point consisted of four or five plants grown together in one pot and exposed to a group of seven DBM larvae. For each treatment and control and for each time point two independent biological replicate experiments were performed and RNA derived from each biological replicate was used for two separate array hybridizations each using reversed fluorescence labels (dye-flip). This experimental design thus resulted in four replicate microarray hybridizations per time point and treatment with two biological and two technical replicates comparing RNA derived from treated plants with the corresponding control harvested in parallel.

**Figure 1:**
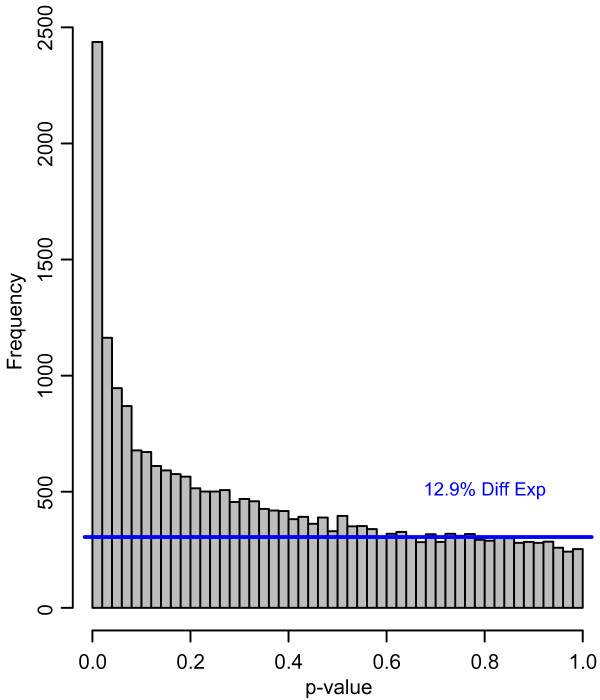
**Distribution of parametric p-values from ANOVA**. For each of the 26,090 probes present on the microarray, normalized expression ratios from four replicate arrays for each of the four time points were used for an analysis of variance (ANOVA). Shown is the frequency distribution of the resulting p-values. A horizontal line indicates the estimated NULL distribution separating the number of true positive tests (above the line) from negative tests within a given p-value bin (falsely discovered genes)

The microarray used in this study is based on a set of 26,090 Arabidopsis gene specific 70-mer oligonucleotides [9]. Upon removal of manually flagged spots, background correction, and flooring, on average 12.5 % of all spots were excluded from further analyses as non-detectible. Signal intensities were used for loess normalization thereby generating log_2_-ratios comparing each treatment with the corresponding control. For each time point, we first used the data from the four replicate arrays to perform a Student's *t*-test and to calculate mean expression ratios for each treatment sample relative to the corresponding control. In order to assess the type I error rate, we calculated *q*-values estimating the false discovery rate based on the parametric *p*-values obtained from the *t*-statistic [17]. We then used the four normalized expression ratios from each of five time points (for a total of 20 data points) to perform an analysis of variance (ANOVA) and again estimated the false discovery rate based on the distribution of parametric *p*-values (Figure 1). Normalized expression ratios for all probes on the array as well as results for all statistical analysis are provided in Additional File 1. As expected, higher *p*-values from *t*-statistics are associated with a higher false discovery rate; for example, after 8 h of herbivory 4,576 probes were characterized with *p*(*t*-test) < 0.05, but have a 11% chance to be falsely discovered (*q *< 0.110), while only 1,476 probes are characterized with *p*(*t*-test) < 0.01, and these have only a 6.8% chance to be falsely discovered (Table 1). However, a relatively large number of array probes were associated with high *p*-values (up to at least 0.08), which still contain a substantial number of truly differentially expressed genes (Figure 1), as estimated from the higher frequency of genes in these *p*-value bins compared to the frequency expected if no genes were differentially expressed (indicated by a horizontal line in Figure 1). Thus, by using a low *p*-value cut-off (0.01), we would reduce the number of falsely discovered genes, but would also miss a substantial number of truly differentially expressed genes. Therefore, assuming that high fold change differences is associated with a lower likelihood of being false positives, we initially defined as 'differentially expressed' (i.e. genes with DBM-induced change in transcript abundance) those genes for each time point that were associated with a *t*-test *p*-value of less than 0.05 (accepting a false discovery rate of up to 0.4) and also displayed a more than two-fold change between treatment and control. Using this definition, the number of differentially expressed transcripts at each time point was found to range from 130 (98 up- and 32 down-regulated) after 1 h of DBM feeding to 1,805 (1,246 up- and 559 down-regulated array elements) after 8 h of herbivory, with a total of 2,881 transcripts that were differentially expressed in at least one time point (Table 1). Among these 2,881 transcripts, 1,854 were significantly up-regulated (designated group A in Additional File 1) while 1,007 were significantly down-regulated (group B) and only 20 were up-regulated at one time point and down-regulated at another (group C in Additional File 1, Table 1). Relatively few genes were differentially expressed at 1 h after the onset of feeding with increasing numbers of differentially expressed genes until 8 h after DBM feeding. Despite ongoing feeding, fewer genes are differentially expressed at 24 h after the onset of feeding. At all time points, much fewer transcripts were down-regulated then up-regulated (Table 1). In summary, a total of 2,881 probes representing the Arabidopsis leaf transcriptome met our strict definition of differential expression in response to DBM feeding in showing a significant (p[t-test] < 0.05) and more than twofold difference of transcript abundance between treatment and control for at least one time point.

**Table 1:** Overall summary of differentially expressed genes

	p(t-test) < 0.01		p(t-test) < 0.05		p(t-test) < 0.05, FC^b^>2			
			
Treatment	genes	max. FDR^a^	genes	max. FDR^a^	genes, total	up	down	mixed
1 h	327	0.589	1350	0.719	130	98	32	-
4 h	524	0.307	2019	0.402	532	407	125	-
8 h	1476	0.068	4576	0.110	1805	1246	559	-
24 h	854	0.173	3124	0.238	1154	658	496	-
In at least 1 time point	2830	-	8471	-	2881	1854	1007	20

	p(ANOVA) < 0.01		p(ANOVA) < 0.05		p(ANOVA) < 0.05, FC^c^>2			
	
Treatment	genes	max. FDR^a^	genes	max. FDR^a^	genes			

All	1756	0.084	4093	0.169	3111^d^			

^a ^the highest expected false discovery rate (FDR); i.e. the maximal q-value observed for the given p(t-test) cut-point

^b ^fold-change between treatment and control

^c ^maximum fold change between treatments [max (treatment/control)/min (treatment/control)]

^d ^of these 3111 genes only 1686 genes are also among the group of 2881 genes that are differentiallyexpressed in at least one time point with p(t-test) < 0.05 and FC^b ^> 2

### Temporal patterns of the Arabidopsis leaf transcriptome affected by DBM

**Figure 2:**
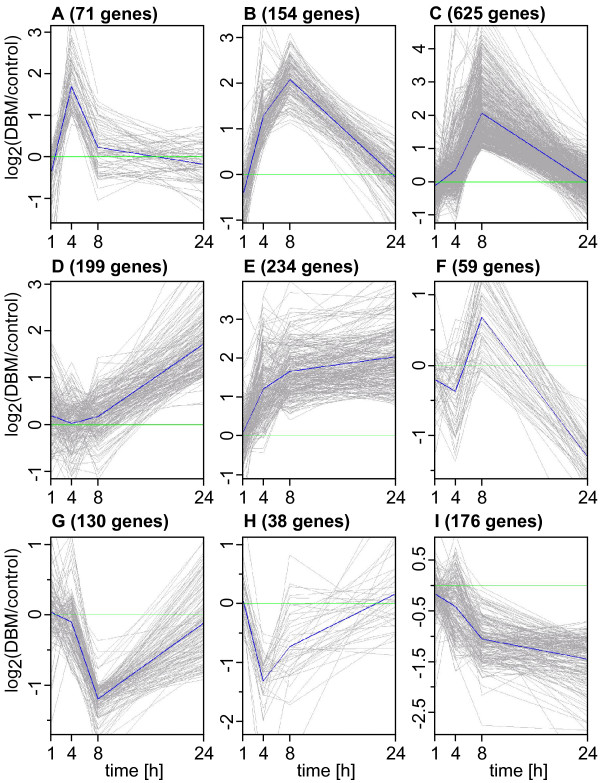
**Expression profiles based on K-means clustering**. Mean log_2_-expression ratios for genes that are differentially expressed in at least one time point (p[t-test] < 0.05 with more than two-fold change of transcript abundance between treatment and control) and which are also changing more than two-fold over time with p(ANOVA) < 0.05 were used for K-means clustering. Each gene within a given cluster is shown with a grey line and the mean expression profile from all genes in a given cluster is indicated with a single blue line. Cluster designation and the number of genes in each cluster are shown above each panel.

To estimate the number of genes that were changing in expression between at least two time points we performed an analysis of variance (ANOVA), and found 3,111 genes that changed more than twofold with *p*(ANOVA) < 0.05 (Table 1). However, of these only 1,686 were also differentially expressed between treatment and control in at least one time point. These 1,686 genes, which met our most stringent definition of differential expression in response to DBM feeding over the 24 hour time course, were placed into nine expression clusters based on their temporal patterns of expression profiles identified by K-means clustering (Figure 2; identification of genes belonging to each cluster is provided in Additional File 1). While 71 genes displayed a rapid transient up-regulation within 1 h upon onset of herbivory (Cluster A), a total of 779 genes peaked at 8 h (cluster B and cluster C). Another group of differentially expressed genes showed up-regulation of transcript abundance mainly at late time-points (cluster D), while 234 genes were up-regulated early during the treatment and maintained high expression levels relative to control plants (cluster E).

Similarly, many down-regulated genes displayed transient expression profiles (cluster G and cluster H), although a majority of down-regulated genes maintained lower expression levels over the time course analyzed (cluster I). Interestingly, a portion of genes placed in the down-regulated clusters displayed reversed expression ratios at different time points, e. g. some genes in cluster H were transiently down-regulated at early time-points but were up-regulated later in the experiment. Likewise, many genes in cluster I were transiently up-regulated early in the treatment, but were down-regulated 24 h after the onset of DBM feeding. In reverse, many genes in cluster F were up-regulated 8 h into the time course, but displayed repressed expression at the 24 h time point. This cluster also contains genes that display a biphasic expression pattern, with repressed expression at early (1 h) and late (24 h) time points.

In summary, it is noteworthy that despite continuous insect feeding over the time period analyzed, a majority (60%) of up-regulated genes displayed a transient pattern of change of transcript abundance.

### Annotation and expression profiles of DBM induced stress-related genes

**Figure 3:**
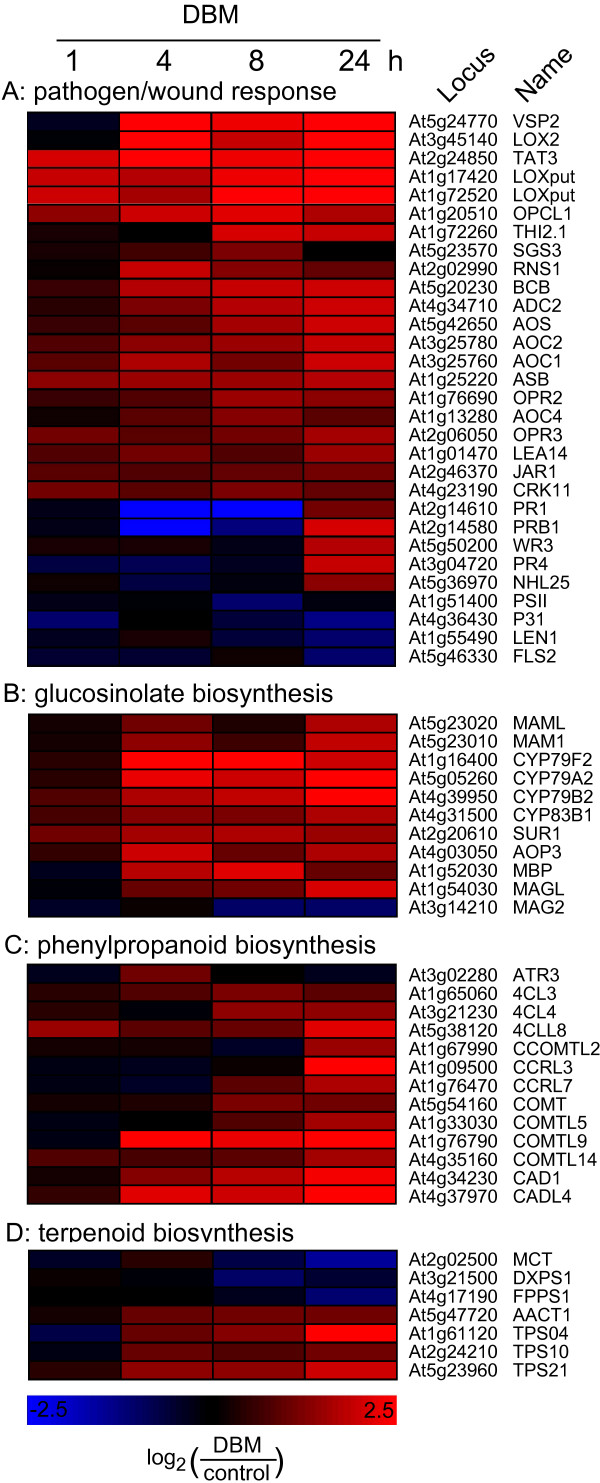
**Expression profiles of defence related genes and genes of secondary metabolism upon herbivory**. (A) A list of curator annotated genes involved in response to pathogens or wounding was retrieved from TAIR [18] Thirty of these genes were differentially expressed in response to DBM feeding in at least one time point and mean expression ratios for these genes were used to generate the heatmap. Bright red indicates a more than 5.7-fold higher level of transcript abundance (expression) in herbivore treated plants compared to control plants; bright blue indicates a more than 5.7-fold lower expression. (B) 22 Arabidopsis genes have been characterized as being involved in glucosinolate biosynthesis [32] and eleven of those were found differentially expressed in at least one time point upon DBM herbivory. These genes were used to generate the heatmap shown here. C) Of 78 genes encoding enzymes of the phenylpropanoid pathway and homologs thereof [9], thirteen were found differentially expressed as shown. Note that only CAD1, 4CL3, and 4CL4 are characterized enzymes while all other represent homologs of known phenylpropanoid genes. (D) Of 229 genes annotated to be involved in Arabidopsis isoprenoid metabolism [11], 30 genes (13%) were differentially expressed upon DBM feeding. Shown as a heatmap are selected genes involved in mono-, sesqui,- or diterpene biosynthesis. For details on expression data and gene name abbreviations search Additional File 1 using the locus identifier provided.

Annotation against higher level GeneOntology terms at TAIR [18,19] revealed that many of the genes up-regulated by DBM feeding fall into the functional categories 'transport', 'response to abiotic or biotic stimulus (stress)', 'protein metabolism', and 'transcription'. In order to analyze expression profiles of stress-related genes in more detail, we retrieved curator annotated TAIR gene lists for the categories 'response to pest, pathogen or parasite' or 'response to wounding' as well as for genes associated with children terms of these categories. We further limited these lists to those genes that have been annotated based on experimental evidence resulting in 150 stress-related genes after removal of duplicates. Of these, 128 were represented on the microarray and 30 (23%) were differentially expressed in response to DBM feeding. Expression data for these genes were used for hierarchical cluster analysis shown with expression maps in Figure 3.

Most of these DBM-affected stress-related genes were strongly up-regulated within 1 to 4 h after the onset of DBM feeding (Figure 3), and the majority were associated with wound response. In contrast, only five genes were up-regulated only at the 24 h time point, including two pathogenesis-related (PR) genes (PR1/At2g14610 and PRB1/At2g14580) that are associated with salicylic acid dependent pathogen defence. These genes are down-regulated early during DBM feeding before being up-regulated at 24 h. Many of the wound-response genes that are strongly up-regulated by DBM are involved in octadecanoid biosynthesis. All known enzymatic steps of the octadecanoid pathway were represented in the cluster of DBM-induced genes (Figure 3A). These include the lipoxygenase LOX2 (At3g45140), two other putative lipoxygenases (At1g17420 and At1g72520), the single copy allene oxid synthase AOS (CYP74A, At5g42650), the allene oxide cyclases AOC1 (At3g2576), AOC2 (At3g25780), and AOC4 (At1g13280), the 12-oxophytodienoate reductases OPR2 (At1g76690) and OPR3 (At2g06050), and 3-oxo-2-(2'-[*Z*]-pentenyl)cyclopentane-1-octanoic acid *C*oA *L*igase1 OPCL1 (At1g20510) [20,21]. Other genes in this group of up-regulated wound-response genes are involved in the shikimate pathway (anthranilate synthase, *ASB*, At1g25220), and tocopherol biosynthesis (tyrosine aminotransferase, *TAT*, At2g24850) [22,23].

We further analyzed expression patterns of genes of secondary metabolite pathways that are known to be affected by herbivory, namely glucosinolate, phenolic and terpenoid metabolism (Figure 3B–D). Many transcripts of these pathways were differentially expressed upon DBM feeding. For the glucosinolate pathway, two genes involved in the chain elongation of methionin (*MAM1 *and *MAML*, At5g23020 and At5g23010) were up-regulated, while two other members of the *MAM *gene family [24] were not detectibly differentially expressed. Also up-regulated were cytochrome P450 monooxygenases involved in the biosynthesis of various glucosinolates: CYP79A2 (At5g05260) catalyzing the conversion of phenylalanine to the corresponding oxime in benzylglucosinolate biosynthesis [25]; CYP79B2 (At4g39950) converting tryptophan and tryptophan analogs to the oxime in indole glucosinolate biosynthesis [26]; CYP79F2 (At1g16400) involved in the synthesis of long chain aliphatic glucosinolates [27]; and CYP83B1 (At4g31500) catalyzing the oxidation of indole-3-acetyldoxime in indole glucosinolate biosynthesis [28]. CYP79B2 is also involved in camalexin and auxin biosynthesis [29,30]. DBM feeding further induced the C-S-lyase (SUR1, At2g20610) that converts S-(alkylacetohydroximoyl)-L-cysteines to the corresponding thiohydroximic acids [31]. While none of the three myrosinase encoding genes present in Arabidopsis [32] were differentially expressed at detectable levels, two myrosinase associated proteins (MAG) were affected. Of these MAG2 (At3g14210), which has been characterized as a quantitative trait locus (termed epithiospecifier 1; ESM1) controlling the ratio of nitrile to isothiocyanate production during glucosinolate hydrolysis [33], was suppressed by DBM feeding, while the related gene MAGL (At1g54030) was induced.

Relatively few genes that have been functionally characterized to encode enzymes of the phenylpropanoid pathway are induced by DBM feeding (Figure 3C), but many of the genes of this pathway just failed the call to be significantly differentially expressed at stringent conditions. In contrast, eight genes with similarity to known phenylpropanoid genes but otherwise of unknown function were transcriptionally up-regulated (Figure 3C).

Related to terpenoid biosynthesis, two differentially expressed genes encoding enzymes of the methylerythritol phosphate pathway, 1-deoxy-D-xylulose 5-phosphate synthase (DXPS1, At3g21500) and 2-C-methyl-D-erythritol 4-phosphate cytidyltransferase (MCT, At2g02500) [11] were down-regulated by DBM feeding. In contrast the only gene of the mevalonate pathway that was detected as affected by DBM feeding encodes acetoacetyl-CoA thiolase (AACT1, At5g47720) and was up-regulated. In addition, a farnesyl diphosphate synthase (FPPS1, At4g17190) and three of the more than thirty Arabidopsis terpene synthase genes (TPS04, At1g61120; TPS10, At2g24210; TPS21, At5g23960) [10] were up-regulated by DBM feeding (Figure 3D).

### Expression profiles of phytohormone related genes affected by DBM feeding

**Figure 4:**
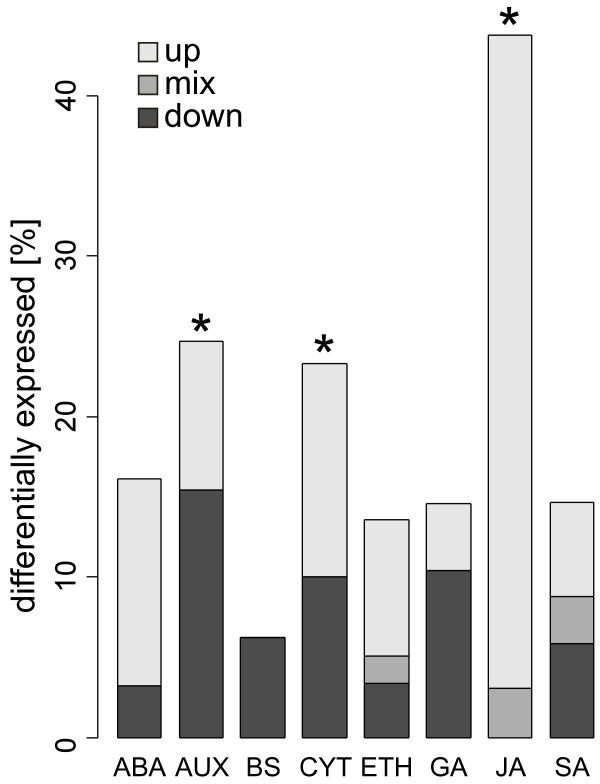
**Differential expression of genes associated with signalling molecules**. Based on curator annotated Gene Ontology categories at TAIR, *A. thaliana *genes were identified that are (i) involved in the metabolism of, (ii) are part of the signal transduction mediated by, or (iii) are responsive to signalling compounds (i.e., ABA: abscicic acid; AUX: auxin; BS: brassinosteroid; CYT: cytokinin; ETH: ethylene; GA: giberellic acid; JA: jasmonates; SA: salicylic acid). Fractions of genes that are differentially expressed upon DBM herbivory (in % of the genes in each category) in at least one time point (p[t-test] < 0.05, fold-change > 2) are shown. The fraction of down-regulated genes is shown in dark grey, the fraction with mixed expression over the time course in medium grey, and the fraction of up-regulated genes in light grey. Stars indicate that the frequency of differentially expressed genes in the given functional group is significantly higher (p[hyper] < 0.05) than the frequency observed in the set of all genes represented on the microarray.

Based on the relatively large number of transcripts up-regulated by DBM that are associated with biosynthesis of octadecanoids (Figure 3), we compared these transcripts profiles with those associated with other signalling molecules and phytohormones, namely salicylic acid, ethylene, auxin, abscisic acid, brassinosteroids, cytokinin, and gibberellic acid (Figure 4). For this purpose, we retrieved TAIR gene lists that had been curator annotated to be either involved in the metabolism of these signalling molecules, to be part of the signal transduction mediated by, or to be responsive to these compounds.

**Figure 5:**
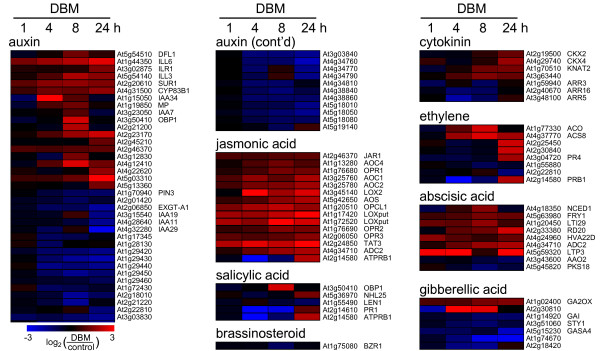
**Expression profiles of hormone related genes affected by DBM feeding**. Based on curator annotated Gene Ontology categories at TAIR, A. thaliana genes were identified that are (i) involved in the metabolism of, (ii) are part of the signal transduction mediated by, or (iii) are responsive to signalling compounds (i.e., ABA: abscicic acid; AUX: auxin; BS: brassinosteroid; CYT: cytokinin; ETH: ethylene; GA: gibberellic acid; JA: jasmonate; SA: salicylic acid). Shown as a heatmap are genes in these categories that are differentially expressed upon herbivory in at least one time point (p[t-test] < 0.05, fold-change > 2). Bright Red indicates a more than 5.7-fold higher transcript abunance in herbivore treated plants compared to control plants; bright blue indicates a more than 5.7 fold lower expression. AGI information and gene names are given on the right, for detailed information on each gene see Additional File 2.

We found that genes associated with the signalling molecules jasmonate, auxin, and cytokinin were significantly over-represented among genes up-regulated by DBM feeding based on a hypergeometric distribution (*p *[hyper] < 0.05) (Figure 4). For comparison, while 11% of all probes on the microarray that are associated with an AGI identifier were differentially expressed upon DBM feeding, 44% of the genes associated with jasmonate biosynthesis, signalling, or response were differentially expressed upon DBM feeding, and all were significantly up-regulated in at least one time point (Figure 4 and Figure 5). In contrast, genes associated with the signal compounds salicylic acid and ethylene were not more frequently differentially expressed than expected for any randomly chosen group of genes. In contrast to jasmonate-related transcriptome signatures, many genes related to salicylic acid and ethylene signalling were transiently down-regulated followed by a late induction during DBM feeding (Figure 4 and Figure 5).

Genes associated with gibberellic acid, brassinosteroids, and abscisic acid were also not over-represented among the differentially expressed genes (Figure 4). However, among those genes that were differentially expressed, most genes associated with abscisic acid were rapidly up-regulated upon herbivory, while genes associated with gibberellic acid were predominantly down-regulated (Figure 4 and Figure 5).

In addition to genes associated with jasmonate signalling, genes associated with the hormones auxin and cytokinin were also over-represented in the DBM affected transcriptome (Figure 4). While three cytokinin oxidase family members involved in cytokinin catabolism were up-regulated late in the feeding experiment, several cytokinin response regulators are transiently down-regulated during herbivory (Figure 5). Likewise, a large group of auxin-induced genes were transcriptionally down-regulated starting at 4 h after the onset of herbivory feeding (Figure 4). However, a smaller group of genes in the same group displayed a reverse expression pattern, as did some genes involved in auxin metabolism and the auxin-response transcription factor *MONOPTEROUS *(Figure 5) [34]. Overall, our results highlight the importance of jasmonate in herbivore induced signalling, and may also suggest roles for cytokinin and auxin as well.

### Expression profiles of genes associated with signal transduction

**Figure 6:**
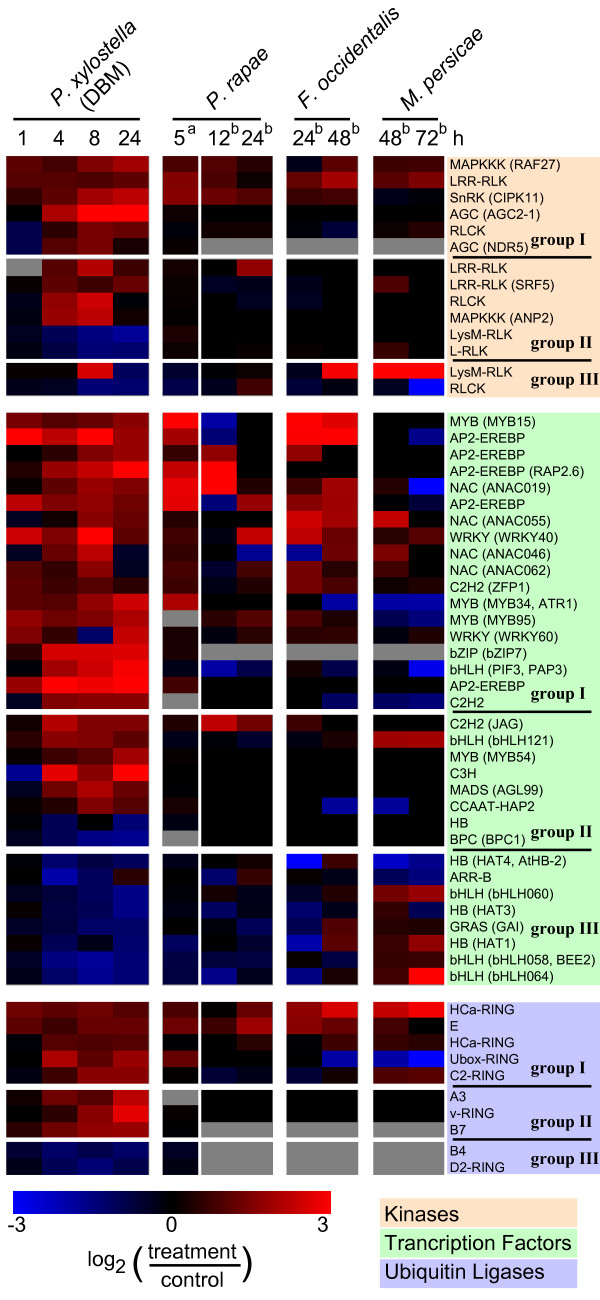
**Gene expression profiles of genes associated with signal transduction components**. Gene information was retrieved from PlantsP (protein kinase families), AtTFDB (transcription factor families), and PlantsUBQ (26S proteasome gene families). 902 protein kinases were present on the array used for DBM herbivory profiling, of which 14 were differentially expressed in at least two time points. 35 transcription factors were differentially expressed in at least two time points among the 1,409 genes represented on the array used. Expression profiles of these genes are displayed as heatmaps: Bright red indicates a more than 5.7-fold higher expression in herbivore treated plants compared to control plants; bright blue indicates a more than 5.7-fold lower expression. Grey indicates missing data. AGI information and sub-family names are given on the right. Gene names are indicated in brackets were applicable. For detailed information on each gene see Additional File 2. The left panel shows results obtained upon DBM treatment (this study). To the right previously published results for the same genes obtained with large scale expression profiling experiments upon herbivory with *P. rapae*, *F. occidentalis*, and *M. persicae *are given (^a^CATMA array platform [3]; ^b^Affymetrix ATH1 array platform [1])

To gain insights into possible signalling processes elicited by DBM feeding, we analyzed expression profiles of genes known or predicted to be involved with signal transduction such as protein kinases, transcription factors, and genes involved in the 26S proteasome pathway (Figure 6). For these genes we retrieved information for complete gene families from PlantsP (protein kinases [35]), AtTFDB (transcription factor families [36]), and PlantsUBQ (26S proteasome gene families [37]).

Of the 902 protein kinases present on the microarray, 98 were differentially expressed, 71 were up-regulated, while 27 were down-regulated (Additional File 2). Fourteen kinases were differentially expressed in at least two time points (Figure 6). Most genes in this group code for receptor-like kinases such as leucin rich repeat (LRR) and peptido-glucan (LysM) binding domain containing kinases. In addition, two mitogen activated protein kinase kinase kinases (MAPKKK) were transcriptionally up-regulated: ANP2 (At3g46160) is a MAPKKK protein related to *Nicotiana *protein kinase 1 (NPK1) which may negatively regulate stress responses [38]; and Raf27 (At4g18950) contains an ankyrin domain but has not been further characterized. A calcium-dependent protein kinase, At2g3036 (CIPK11, SnRK3.22) which is a member of a plant specific protein kinase family that specifically interacts with the calcium sensor protein CalcineurinB-like [39], was also up-regulated by DBM feeding. Finally, two AGC kinases (protein kinases A, G, and C), which belong to a family of effectors of the intracellular second messengers cAMP, cGMP, phospholipids, and Ca^2+ ^[40], were up-regulated upon herbivory.

**Figure 7:**
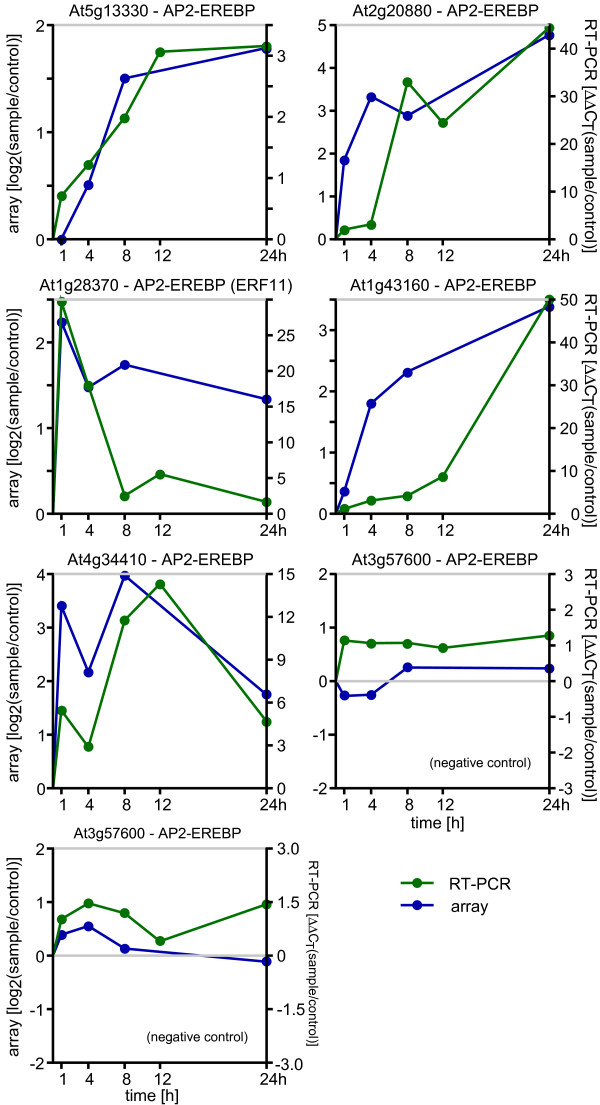
**Comparison of qRT-PCR and microarray expression data for AP2-EREBP genes**. The five AP2-EREBP genes found up-regulated in the microarray data (see Figure 6) and two AP2-EREBP genes that were not significantly differentially expressed as detected on the microarray were tested for RNA abundance using quantitative real time RT-PCR. As an internal standard, expression levels of At5g62700 (TUB, tubulin) was used to determine relative expression levels in each sample (ΔCT values). Shown in green are the ΔΔCT values comparing ΔCT values of DBM treated samples with untreated control samples at 1 h, 4 h, 8 h, 12 h, and 24 h of continuous feeding (right scale bar). For comparison, normalized log2-expression ratios found using the microarray platform are given in blue (left scale).

Among the 1,409 transcription factors represented on the array, 173 were differentially expressed in at least one time point, with 118 being up-regulated and 53 down-regulated, while two displayed a mixed expression (Additional File 2). Thirty five transcription factors were differentially expressed in at least two time points, ten of which were down-regulated and 25 were up-regulated (Figure 6). Among the ten down-regulated transcription factors, basic helix loop helix (bHLH) and homeodomaine binding (HB) proteins of the HD-ZIP II class form the dominant group. Transcription factors that were up-regulated by DBM feeding predominantly belong to AP2-EREBP, MYB, and NAC type factors (Figure 6). Notably, three MYB and three AP2-EREBP factors were up-regulated rapidly within 1 h after the onset of DBM feeding and stayed up-regulated. Expression of all five AP-EREBP transcription factors found to be DBM-induced by microarray analysis also showed highly similar expression profiles when validated by qRT-PCR (Figure 7). Although several AP2-EREBP type transcription factors have been associated with regulating stress responses, to date none of the transcription factors identified here has been characterized in detail. Among the DBM-induced MYB transcription factors, only MYB34/ATR1 has previously been characterized as a positive regulator of indole glucosinolate biosynthesis [41], which is consistent with results that most of its target genes were up-regulated by DBM feeding (Figure 3, Additional File 2).

Targeted protein degradation via the ubiquitin/26S-proteasome pathway is another important regulatory process [42]. Among the 1,403 Arabidopsis genes annotated to be involved in this pathway, 1,230 are present on the array and 82 were differentially expressed upon DBM feeding (Additional File 2). Among these are a 75 putative E3-ubiquitin-protein-ligases that were affected by DBM, in addition to five differentially expressed 26S-proteasome components, a single ubiquitin-like gene and a E2-ubiquitin activating enzyme (UBC10, At5g53300) (the latter two were down-regulated). Figure 6 shows the expression profile of ten different E3-ubiquitin-protein-ligases that were differentially expressed in at least two time points. In summary, we identified a large number of signal transduction components affected by DBM feeding. In particular, members of the AP2-EREBP family of transcription factors stand out as being rapidly induced by herbivory suggesting roles for this family in DBM induced signal transduction networks.

## Discussion

Infestation by feeding DBM larvae induces substantial overall changes in the Arabidopsis leaf transcriptome, with 1,854 array elements representing 1,664 annotated genes that were significantly induced and 1,007 elements representing 913 annotated genes that were repressed significantly. Despite continuous feeding the majority of differentially expressed genes displayed a transient expression profile with a maximum transcript abundance level at 8 h after onset of feeding and were down to their initial transcript level after 24 h (clusters B and C, Figure 2). It is not known what proportion of the induced changes of the transcriptome result in any downstream changes; and it is possible that some defence responses only require a transient burst at the level of the transcriptome to become effective and a large part of the initial response could return to the pre-attack steady-state level of gene expression. Repressor proteins, such as the recently discovered JAZ proteins involved in the mediation of jasmomate signalling [13,14], may be involved in shaping such rapid and transient responses.

Although only a few plant species have been studied for the impact of insect attack on large-scale transcriptome changes, their diversity ranges from relatively short-lived herbaceous plants to long-lived trees, including angiosperms and gymnosperms. Results obtained with these systems support the general notion that insect feeding induces massive changes of the host plant transcriptome [1-6,43,44]. A few general patterns have emerged from these studies suggesting that herbivory can results in down-regulation of primary metabolic processes while at the same time activating defence related processes including secondary defence metabolism. These findings are well supported by our analysis of the Arabidopsis transcriptome affected by DBM feeding. In addition, the massive reprogramming of primary and secondary metabolic processes as part of the insect-induced defence response involves rapid changes in signalling and other regulatory processes. The present study establishes a signature of DBM-induced changes of the signalling transcriptome of Arabidopsis leaves.

**Figure 8:**
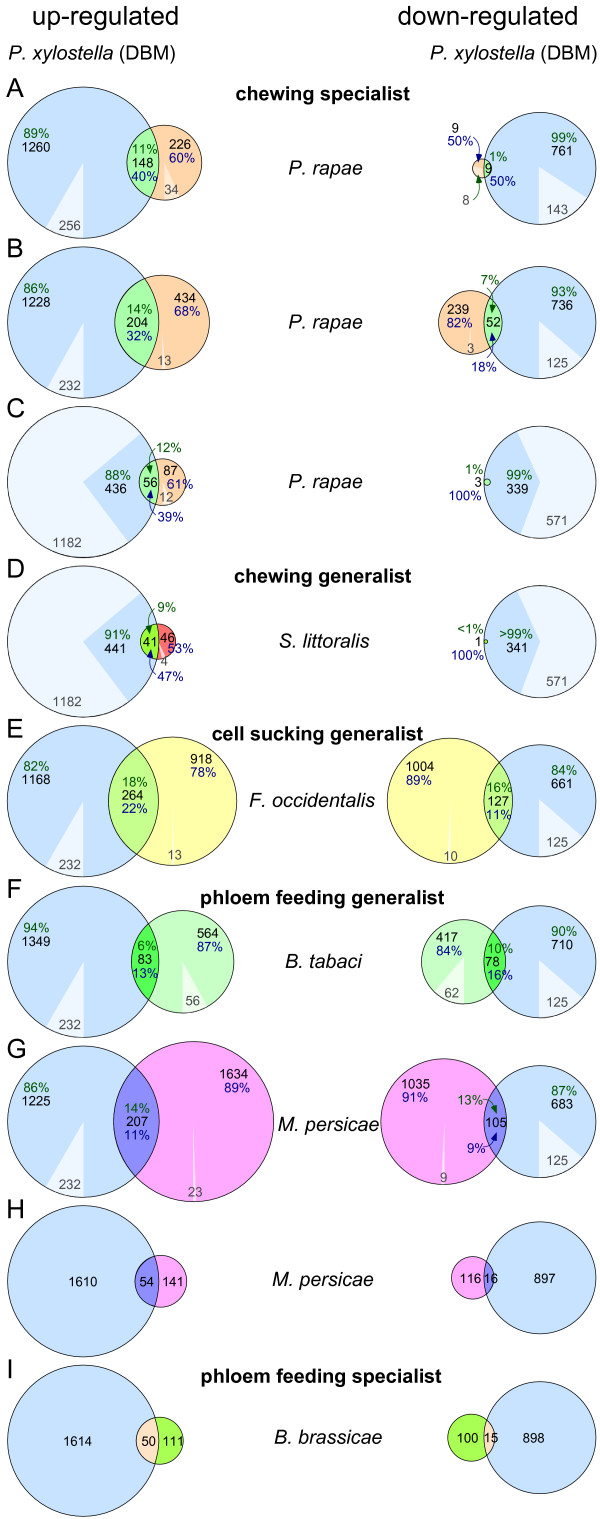
**Venn diagrams comparing the DBM induced transcriptome changes with the response to other herbivores**. The number of induced (up-regulated, left column) and repressed (down-regulated, right column) genes upon DBM feeding (indicated by blue circles) was compared with the response to other herbivory treatments that have previously been published [1-3,5,15]. Circle areas are drawn to scale to the number of genes found differentially expressed (DE). Given with absolute numbers and in percent is the fraction of differentially expressed genes that is regulated in the same overall manner in the two datasets of comparison (overlapping area), or that is affected only by DBM or only by the other herbivore. In A to G the fraction of differentially expressed genes found in a given experiment that is not present on the other array is shaded in lighter colours. For H and I the necessary microarray platform information for a detailed comparison are unpublished. If more than one time point or more than one ecotype were used, a gene was defined as DE if it was up- or down-regulated in at least one of the different treatments with the same herbivore using the same array platform. A) Herbivore: *Piris rapae*; Arabidopsis ecotype: Col; time point: 5 h; definition of DE: p(t-test) < 0.05 & foldchange > 2 (replicates: n = 7); platform: CATMA array, unique AGI loci: 15,722 [3]. B) *P. rapae*; Col; 12 h or 24 h; DE: 'present' in at least one array & foldchange > 2 (n = 1); Affymetrix ATH1, unique loci: 21,833 [1]. C) *P. rapae*; Col; 3 h to 5 h, 24 h local, or 24 h distal; DE: p(t-test) < 0.05 & foldchange > 2 (n = 5); platform: cDNA array, unique AGI loci: 7,200 [5]. D) *Spodoptera littoralis*; Col; 3 h to 5 h; DE: p(t-test) < 0.05 & foldchange > 2 (n = 5); platform: cDNA array [5]. E) *Frankliniella occidentalis*; Col; 12 h or 24 h; DE: 'present' in at least one array & foldchange > 2 (n = 1); platform: Affymetrix ATH1 [1]. F) *Bemisia tabaci*; Col; 21d; DE: SAM *q *< 3.917% & foldchange > 2 (n = 2); platform: Affymetrix ATH1 [2]. G) *Myzus persicae*; Col; 48 h or 72 h DE: 'present' in at least one array & foldchange > 2 (n = 1); platform: Affymetrix ATH1 [1]. H) *M. persicae*; Ws, Cvi, or Ler; 1d; DE: q(t-test) < 0.05 & foldchange > 2 (n = 4); oligo-array, unique AGI loci: 2,158 [15]. I) *Brevicoryne brassicae*; Ws, Cvi, or Ler; 1d; DE: q(t-test) < 0.05 & foldchange > 2 (n = 4); oligo-array [15]. Data for all pairwise comparisons as well as the numbers for multiple intersects are given in Additional File 3. This table also contains information on all genes being called DE in at least one experiment.

In order to more broadly identify common patterns of the transcriptome response of Arabidopsis to different herbivores, we compared data obtained in this study with other large scale expression analyses that used different array platforms to study the transcriptome responses induced by *P. rapae *[1,3,5], *S. littoralis *[5], *F. occidentalis *[1], *B. tabaci *[2], *M. persica *[1,15], and *B. brassicae *[15] (Figure 8; see figure legend for details on the platforms used and the time points analysed). Despite the large differences in the biological materials and the different array platforms used (in particular the coverage of the array platforms varied greatly), which precludes complete comparisons, a substantial overlap in the groups of up- or down-regulated genes was apparent (Figure 8). The highest relative degree of overlap of the DBM-affected transcriptome was found with the effect of *S. littoralis*, as DBM a leaf chewing caterpillar, with 47% (41 genes) of differentially expressed genes common on both platforms being induced by both *S. littoralis *and DBM. Similarly, between 32% and 40% (56 to 204 genes) of those genes that are in common to the respective platforms used in studies with *P. rapae *and in our analysis with DBM agreed in their general induction (Figure 8). Both *P. rapae *and DBM are leaf chewing caterpillar specialized to the *Brassicacea*. Overall lower degrees of relative overlap in the group of induced genes were found when the response induced by DBM was compared with the responses induced by cell-sucking thrips, *F. occidentalis*, or the phloem sap-feeding herbivores *M. persicae*, *B. tabaci*, and *B. brassica *with 11% to 31% of induced genes found in common. In this comparison, the highest relative degree of overlap was found with the aphid *B. brassica*, which is as DBM a specialist herbivore. Together these comparisons suggest that herbivores with a similar mode of feeding may induce a more similar transcriptome response in the host.

Surprisingly, in the meta-analysis of all microarray data represented in this comparative Arabidopsis-herbivory transcriptome study (Figure 8 and Additional File 3), we found only one gene being up-regulated in nine of the ten experiments compared: The cytochrome P450 monooxygenase CYP79B2, which catalyzes the conversion of tryptophan to indole-3-acetaldoxime, the precursor of indole glucosinolates, camalexin, and also auxin [26,29,30,45]. Six additional genes were found up-regulated in eight of the ten experiments, including three other genes related to tryptophan metabolism and glucosinolate biosynthesis as well as the jasmonate inducible tyrosine aminotransferase TAT, which is involved in tocopherol biosynthesis [22,46]. An additional 40 genes were found induced in at least six of the experiments compared (Additional File 3), and almost half of these (18 genes) were found in the GeneOntology category 'response to stress' including genes encoding enzymes of the shikimate pathway (in particular the tryptophan branch), phenylpropanoid metabolism, glucosinolate bioynthesis, glutathione metabolism, and chlorophyll degradation (Additional File 3). Genes encoding functions in the octadecanoid pathway were also found induced in most of the individual datasets of this comparative Arabidopsis-herbivory transcriptome meta-analysis. The role of octadecanoids in mediating herbivore-induced responses is well established, and it has been estimated that up to 80% of all herbivore-induced Arabidopsis genes are octadecanoid regulated [5].

**Figure 9:**
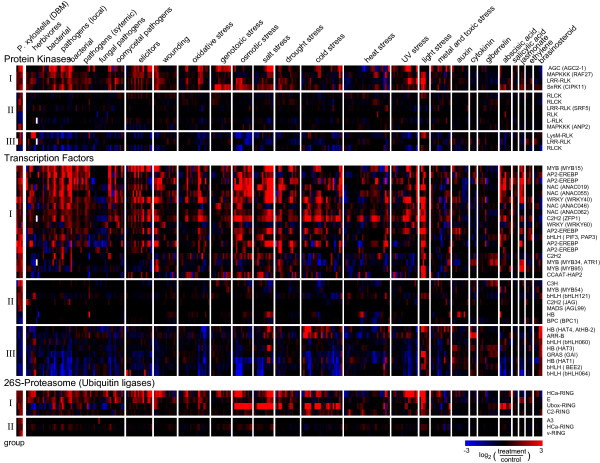
**Meta-analysis of DBM-affected signalling elements**. Expression data (normalized intensities) based on publicly available Affymetrix microarray hybridization data were retrieved from the Genevestigator database [55]. All probe sets called absent were set to the mean intensity of these undetectable probe sets for each gene. If replicate experiments were performed the mean intensity of all replicates was calculated. Expression ratios for all treatment experiments were generated using the corresponding control experiments and the log_2 _transformed ratios were used to generate a heatmap. Each column represents one experiment; each row represents one of the candidate genes as indicated to the right. Bright Red indicates a more than 5.7 fold higher expression in treated plants compared to control plants; bright blue indicates a more than 5.7 fold lower expression. Brief descriptions of the experimental treatments are given on top. Details on each experiment are given in Additional File 4.

Our analysis of the DBM-induced response in Arabidopsis agrees with the central role of ocatdecanoids since jasmonate related genes were significantly over-represented in the set of DBM-induced genes. While auxin and cytokinin related genes also appear to be involved in different aspects of the responses to DBM, in contrast to the jasmonate related genes, a substantial portion of the cytokinin and auxin related genes were down-regulated (Figures 4 and 5). Cytokinin and auxin are important in the control of plant morphogenesis and frequently act antagonistically [47-49]. Cytokinin is known to promote cell division, delays leaf senescence and may have a role in reallocation of resources from source to sink tissues [50]. In previous studies, auxin levels were found to be decreased in tobacco and maize after wounding or upon herbivore infestation [51,52], and external application of auxin decreased wound responses including production of jasmonate [53] and proteinase inhibitor gene expression [51]. Auxin related genes were also found to be down-regulated in wounded Arabidopsis plants [54]. Thus, auxin- and jasmonate-dependent processes may be subject to opposite regulation in the plant response to wounding or herbivory. In support of this notion, we found that many of the stress-related signal transduction components induced upon DBM herbivory are also induced by methyl jasmonate, but are repressed by auxin (Figure 9).

In the present study we identified a large number of transcripts that are affected by DBM feeding and are broadly associated with signal transduction components (examples are highlighted in Figure 6 and the complete data set is provided in Additional File 2). Transcriptome patterns associated with signalling in insect attack have not been well established. In other plant species this is largely due to the smaller array platforms commonly available that may not include many transcription factors or other signalling features. Also, lack of relevant reference datasets such as those obtained from array analyses of other stress treatments in the same plant species make meta-analyses of signalling transcriptomes currently a difficult task for most plant species, except for Arabidopsis. We established a first meta-analysis of DBM-induced regulatory proteins (protein kinases, transcription factors, ubiquitin ligases) to integrate results obtained in the present microarray analysis of DBM-induced Arabidopsis leaves with previously published Arabidopsis microarray data from various treatments related to biotic and abiotic stress response (Figure 9, Additional File 4). Specifically, we selected microarray data from a total of 295 Arabidopsis samples treated with a wide range of biotic and abiotic stresses, phytohormones and biological elicitors, or other chemicals (Figure 9, Additional File 4). The data analyzed in this comparison are based on the Affymetrix ATH1 platform, were retrieved from the 'Genevestigator' database [55], and were processed as described in Additional File 4. Based on this meta-analysis, the DBM-affected transcription factors and other signalling components identified in our experiments can be divided into two large classes: (i) those that are responsive to a wide range of different forms of biotic and abiotic stress [Figure 9, group I (induced) and group III (repressed)]; and (ii) those that are not effected by most other treatments of this comparison (Figure 9 and Additional File 4, group II).

Signalling components in group-I and group-III thus constitute candidates involved generally in stress perception and transduction. Group-I and group-III transcription factors include all DBM-affected AP2-EREBP genes, the bHLH *PIF1*, *WRKY80*, *WRKY40*, the C2H2 *At5g04340*, *MYB15*, and all DBM-effected NAC type transcription factors. Most of these genes were also found to be induced by other herbivores, although with notable exceptions (Figure 6). For example, the AP2-EREBP At2g2088 is rapidly and strongly induced by DBM feeding, but appears unaffected by the other herbivore treatments, while treatments with some bacterial or fungal pathogens, drought, and abscisic acid lead to transiently induced expression (Additional File 4).

Signalling components in group-II appear to be specific to DBM treatment, as they were not found induced in most other stress treatment (Figure 9). However, lack of induction in other treatments needs to be interpreted cautiously, since different expression profiling platforms were used. The transcription factors JAG (At1g68480) and bHLH121 (At3g19860), which are members of group II, were also found induced upon herbivory with *P. rapae *and *M. persica*e, respectively (Figure 6) but were found induced in only three and five, respectively, of the other 297 stress treatments (Figure 9 and Additional File 4). JAG had previously been shown to be necessary for the development and shaping of lateral organs such as leaves [56]. Taken together, we identified a large set of signal transduction components that likely orchestrate a rapid and general response to a wide range of external stresses, but also a large set of signaling components that may mediate responses more specific to plant-herbivore responses.

Very few of the protein kinases, transcription factors and ubiquitin ligases that are affected by DBM feeding (Figure 9, Additional File 4) have well characterized functions. A notable exception is the transcription factor MYB34/ATR1 (At5g60890), which encodes a positive regulator of indole glucosinolate biosynthesis. ATR1 has previously been implied in insect-induced signalling [41] and is induced in particular late into the DBM feeding experiment. ATR1 was also found induced upon herbivory by *P. rapae *[3], but appears repressed upon treatment with *F. occidentalis *and *M. persicae *[1] (Figure 6). Three additional transcription factors, the two AP2-EREBP *RAP2.6 *(*At1g43160*) and *At2g20880 *as well as *MYB15 *(*At3g23250*), were previously identified as induced by wounding, methyl jasmonate, various pathogens, and several forms of abiotic stress [57].

## Conclusion

The Arabidopsis transcriptome changes substantially in response to leaf feeding DBM larvae. Major DBM induced changes are involved in specialized (secondary) defence metabolism and in signalling. The DBM induced response shows considerable overlap with the response induced by other insect herbivores. A first large-scale meta-analysis of Arabidopsis microarray data obtained for a large number of biotic and abiotic interactions revealed groups of transcription factors and other signalling components that are similarly affected by multiple forms of biotic or abiotic stress including DBM feeding or, alternatively, appear more specifically responsive to DBM herbivory.

## Methods

### Plant and insect materials

*Arabidopsis thaliana *plants (ecotype *Landsberg erecta*) were grown in plastic pots (10 cm wide × 8 cm tall) containing Terra-lite Redi-earth (W.R. Grace and Co., Ajax, Ontario, Canada). Each pot contained four or five plants, which were grown in a growth chamber at 20°C constant temperature, 8 hr/16 hr Light/Dark photoperiod at 50–60% ambient humidity, for 8 to 9 weeks. Short day conditions prevented the onset of flowering and the plants were thus maintained in growth stage 1 (leaf production) with 13 to 15 rosette leaves larger than 1 mm (stage 1.13 to 1.14). Diamondback moth (DBM, *Plutella xylostella*) larvae were provided by Dr. Murray Isman (Faculty of Agricultural Sciences, University of British Columbia, Vancouver, Canada) and maintained on cabbage (*Brassica oleracea*) plants in a climate-controlled room at 25°C, 12 hr photoperiod with 50%-60% relative humidity. Two days before exposing *A. thaliana *plants to herbivore treatment, plants were transferred to a climate-controlled room (22°C, 50–60% humidity, 12 hr photoperiod). For insect treatment, seven DBM larvae (third to fifth instars) were placed on a group of four or five plants until time of harvest, for each time point separately. As control, Arabidopsis plants were maintained under the same condition except without exposure to DBM larvae. Rosette leaves from DBM-exposed and control plants were harvested at 1 h, 4 h, 8 h, 12 h and 24 h after onset of herbivory. For each treatment group and time point, all rosette leaves were harvested from the four or five plants per treatment group and flash frozen in liquid nitrogen.

### Microarrays, RNA isolation, cDNA labelling, and microarray hybridization

The design and production of the *A. thaliana *26,090 element 70-mer oligonucleotide microarray was previously described with oligonucleotides designed in collaboration with and purchased from Operon (Huntsville AL, USA) [9]. All procedures for RNA isolation, RNA labelling and microarray hybridization were performed as described [9]. Microarray experiments involved two independent biological replicates for each time point and treatment with each replicate consisting of four or five plants to provide enough plant material for RNA isolation. In addition, microarray hybridizations for each time point and treatment were performed with two technical replicates (dye-flip labelling) for each of the biological replicates for a total of four dual channel microarray hybridizations per time point comparing treatment with control.

### Microarray data analysis

Microarrays were scanned with a ScanArray Express (Perkin Elmer, Woodbridge ON, Canada) scanner with laser power set to 95% and photo-multiplier-tube set to 54 to 64. We identified and quantified hybridization signals using ImaGene software (BioDiscovery, Marina Del Rey CA, USA). Grids were manually placed and spot finding was performed using the 'Auto adjust' spot function repeated three times. Spot finding was subsequently verified by visual inspection and manually adjusted when necessary. Poor spots were manually flagged (flag 1) and were not used in further data analyses. For all analyses, the median pixel intensities for each spot were used. All microarray expression data were submitted to the GEO database [58] under the accessions series GSE10681. Further analyses were performed using customized scripts for R and Bioconducter [59]. For background correction, we defined the mean of the lowest 10% of spot intensities from a particular subgrid as the background for that subgrid. This mean was subtracted from each spot in the subgrid. Signal intensities that did not exceed the background plus 3 standard deviations thereof were defined as not detectable and were excluded from further analyses. We normalized using loess curves [60]. For each array element, we first used the data from the four replicate array hybridizations (two biological replicates each with two technical replicates) for each time point and treatment to perform a paired Student's t-test using the Welch approximation to degrees of freedom. Subsequently, an analysis of variance (ANOVA) using data from all experimental samples (four normalized log_2_-expression ratios per time point for a total of 20 data points) was performed for each element. In order to assess the type I error rate, we calculated q-values estimating the false discovery rate based on the parametric p-values [17]. Genes were first placed into one of three expression groups: Group A) up-regulated genes displaying a significant (p[t-test] < 0.05) and more than twofold higher signal in insect treated leaves compared to control leaves in at least one time point; Group B) down-regulated genes displaying a significant (p < 0.05) and more than twofold lower signal in insect treated compared to control plants in at least one time point; and Group C) genes with mixed expression using the same definition as in A and B. For clustering, mean log_2_-expression ratios for genes identified as differentially expressed (DE) in at least one time point were used. To derive at a reliable dataset, we defined genes as DE only if they met all of the following criteria: (1) significant (p[t-test] < 0.05) and more than twofold difference of transcript abundance between treatment and control for at least one time point, and (2) change of expression of more than twofold between at least two time points of the treatment time course with p(ANOVA) < 0.05. K-means clustering of DE genes was performed using Genesis v1.5 [61] defining nine clusters with a maximum of 50 iterations. The normalized expression ratios and the results for all statistical analyses are summarized in Additional File 1.

### Analyses of genes of interest

Gene lists containing selected genes of interests were retrieved from 'The Arabidopsis Resource Information database' (TAIR) [18] or from published gene family compilations. Lists of genes involved in "*response to pathogens or wounding*" were retrieved from TAIR (status December 2004). We selected genes placed in the GeneOntology (GO) categories "*involved in*" the "*biological process*" "*response to pest, pathogen, or parasite*" and/or "*response to wounding*". Because of the large number of genes of uncharacterized functions associated with the GO-terms, we only selected genes if they were also curator annotated based on experimental evidence by TAIR. Children terms of these GO categories were also included in the selection of these genes. Lists of complete gene families involved in "*Arabidopsis secondary metabolism of glucosinolates, phenylpropanoids, or terpenoids*" were compiled based on published surveys of the Arabidopsis genome [9-11,32]. Complete lists of putative "*protein kinases*, *transcription factors*", and genes involved in the "*26S proteasome pathway*" were retrieved from the PlantsP (protein kinase families [35]), AtTFDB (transcription factor families [36]), and PlantsUBQ (26S proteasome gene families [37]) databases, respectively. For analysis of genes associated with "*phytohormones or signalling compounds*", curator annotated genes placed in the GO terms "*involved in the metabolism of*", "*involved in the signalling mediated by*", or "*involved in the response to*" the phytohormones or signalling molecules "*auxin*", "*abscisic acid*", "*brassinosteroid*", "*cytokinin*", "*ethylene*", "*gibberellic acid*", "*jasmonic acid*", and "*salicylic acid*" were retrieved from TAIR. Each of these gene lists was filtered to avoid multiple entries per list of the same gene.

Expression data for members of these gene lists that were found differentially expressed in our experiments according to the DE definition described above were visualized as heatmaps using Genesis v1.5 [61]. To assess if any of the groups of genes associated with phytohormones or signalling compounds was significantly overrepresented in the insect-effected Arabidopsis transcriptome, the frequency of differentially expressed genes associated with each of the different phytohormones or signalling compounds in the groups A, B, and C (see above) was compared to the frequency of all genes associated with these categories represented on the microarray using a hypergeometric distribution. Phytohormone or signalling compound GO groups were defined as significantly over-represented in the group of differentially expressed genes, when p[hyper] < 0.01.

### Quantitative real time RT-PCR (qRT-PCR)

Total RNA (15 μg) was digested with 15U DNAse in 1 × buffer (Invitrogen, Carlsbad CA, USA) for 15 min at room temperature. The reaction was stopped with EDTA (2.3 mM final concentration) and heat-inactivation (65°C, 10 min). RNA was precipitated with a 1/10 volume of 3 M sodium acetate and 2.5 volumes of ethanol at -20°C overnight, and subsequently pelleted at 20,000 × *g *for 30 min at 4°C. The precipitate was washed with 70% ethanol, centrifuged, and resuspended in RNAse free water to an approximate concentration of 1 μg/μl. Actual RNA concentration was determined spectrophotometrically. DNAse-treated total RNA (10 μg) of was used for reverse transcription with 0.27 μM T_17_VN primer, 0.15 mM dNTPs, 40 U RNAseOut, and 400 U SuperscriptII (Invitrogen) in 10 mM DTT and 1 × first strand buffer in a total volume of 40 μl. Prior to addition of enzymes the solution was heated to 65°C for 5 min and for primer annealing cooled to 42°C. Following an incubation at 42°C for 2.5 h, the RNA was degraded with 8μl 1 M sodium hydroxide for 15 min at 65°C, neutralized with 8 μl 1 M hydrochloric acid and buffered with 4 μl 1 M Tris-pH 7.5. Synthesized cDNA was purified using the Quiagen (Hilden, Germany) PCR-purification kit prior to quantitative PCR reaction using the ABI TaqMan system. The Custom TaqMan Gene Expression Assays (consisting of gene-specific TaqMan^® ^MGB probe and primer sets, supplied as 20× stocks) used for quantitative real time PCR were from the Applied Biosystems (Foster City CA, USA) Custom Oligonucleotide Synthesis Service. The gene-specific probe and primer sets were designed using the Primer Express software from Applied Biosystems. Oligonucleotide sequences of all primer pairs and the respective probes are given in Additional File 5. Multiplex PCR reaction in triplicate (20 μl) containing cDNA equivalent to 100 ng RNA were performed in 96-well plates with the Opticon 2 (BioRad, Hercules CA, USA) using 1 μl of the 20× Custom TaqMan Gene Expression Assay (consisting 900 μmole of primer/250 μmole FAM-labeled probe, final concentration) for each of the genes analyzed; 2 μl primer pair and probe combination (300 μmole of primer/125 μmole of VIC-labeled probe) of the endogenous control (β-tubulin); 10 μl of 2× TaqMan Universal PCR Master Mix (containing all necessary components for fast reaction set-up for 5' nuclease reactions, including AmpliTaq Gold DNA polymerase, and AmpErase UNG). After an initial hold at 50°C for 2 min for the activation of AmpErase UNG, and denaturing at 95°C for 10 min, 40 cycles at 95°C for 15 sec and 60°C for 1 min, followed by fluorescence reading were performed. Data analysis was done according to a protocol by Applied Biosystems. Briefly, the baseline was set such that the amplification curve growth began at a cycle number that was greater than the highest baseline number. The threshold value was set within the exponential phase of the logarithmic scale amplification plot. Relative quantification of gene expression was calculated from the threshold cycle (C_T_) values for each replicate well on the reaction plate. Relative gene expression levels were calculated manually from the exported results file. Briefly, the VIC C_T _values were subtracted from the FAM C_T _values to calculate ΔC_T _for the control and samples at each of the time points for each of the transcription factors [ΔC_T _= C_T (FAM) _- C_T (VIC)_]. The ΔC_T _values for the triplicate wells of the Control samples at each time point for each of the transcription factor were averaged to obtain the mean ΔC_T (Control)_. The mean ΔC_T (Control) _for a gene at a particular time point was then subtracted from the ΔC_T _values of this gene at that time point to calculate its ΔΔC_T (Sample) _[ΔΔC_T (Sample) _= ΔC_T (Sample) _- mean ΔC_T (Control)_]. The ΔΔC_T (Sample) _values were then averaged for the triplicate wells of each gene at each time point to normalize the target mRNA quantity to the internal control (β-Tubulin). The Average ΔΔC_T (Sample) _for each sample was then used to calculate the relative quantification values [2^-mean ΔΔC^_T_].

## Authors' contributions

JE performed and directed experiments, analyzed microarray data, and performed database analyses. SGC performed qRT-PCR experiments and analyzed data. NM assisted with microarray hybridizations. DSA supported statistical analyses. GIA performed herbivore treatments. JB conceived the study, directed experiments, and analyzed data. JE and JB wrote the manuscript. All authors read and approved the final manuscript.

## Supplementary Material

Additional file 1 **Normalized expression data of all non-control elements upon herbivory with DBM**. Arabidopsis L*er *plants were challenged with *P. xylostella *(DBM) larvae for 1 h, 4 h, 8 h, and 24 h. Untreated control tissues were harvested in parallel. The experiment was repeated twice (biological replicates) and RNA from each treatment/control sample pair was used twice (dye-flip replicates) for hybridisation of a printed 70-mer oligonucleotide microarray containing 26,090 non control elements. Given for all non-control elements are information on the genes recognized (columns A to D), the normalized expression ratios for each hybridization (columns Y to AN), the mean ratios from the four replicates (columns L to O), clustering results (columns H and I), and the results of statistical analysis as indicated (columns F, G, J, K, P to W). Ratio considered significant (p[t-test] < 0.05 & foldchange > 2) are indicated in red (induced) or blue (repressed). Click here for file

Additional file 2 **Expression data of genes related to signalling components**. **Sheet a**: Expression data of hormone related genes affected by DBM feeding. Based on curator annotated Gene Ontology categories at TAIR (colums D and U), A. thaliana genes were identified that are (i) involved in the metabolism of, (ii) are part of the signal transduction mediated by, or (iii) are responsive to signalling compounds (i.e., abscicic acid; auxin; brassinosteroid; cytokinin; ethylene; gibberellic acid; jasmonate; salicylic acid). Shown are genes in these categories that are differentially expressed upon herbivory in at least one time point (p[t-test] < 0.05, fold-change > 2). **Sheet b**: Expression data of DBM responsive signal transduction components (protein kinases, transcription factors, and 26S proteasome components). Complete gene family information were retrieved from PlantsP (protein kinases [34]), AtTFDB (transcription factor families [35]), and PlantsUBQ (26S proteasome gene families [36]). Shown are expression data for genes found differentially expressed (p[t-test] < 0.05 and more than twofold change) in at least one time point. Click here for file

Additional file 3 **Venn analysis of differentially expressed genes**. To the left the number of communalities in the set of differentially expressed genes comparing DBM responses with previously published large scale transcriptome analyses. References are given in the title of each experiment. a and b denote the sets being intersected, with b being the last set to be intersected. First pairwise intersects are shown, then thresome intersects, etc. This comparison is based on a summary of experimental sets, i.e. a gene was counted as differentially expressed (DE) if at least one sample in a given experimental series was considered DE (see Figure 8 for definitions of DE). A list of all genes being considered as DE is given to the right. First, the maximum fold change in any of the indicated samples is given; ratios are given only if at least one treatment was called DE. Second, ratios for all time points in each experiment are given; ratios are shown only if the gene was called DE. **Sheet a**: induced genes; **sheet b**: repressed genes. Click here for file

Additional file 4 **Meta-analysis of DBM-affected signalling component**. Expression data (normalized intensities) based on publicly available Affymetrix microarray hybridization data were downloaded from the Genevestigator database [54]. All probe sets called absent were set to the mean intensity of these undetectable probe sets for each gene. If replicate experiments were performed the mean intensity of all replicates was calculated. Expression ratios for all stress related and hormone treatment experiments available were generated using the corresponding control experiments and the log_2 _transformed ratios were used to generate a heatmap. On top of each column the name, description, and the Affymetrix probeset used are indicated. Brief descriptions of each experiment and, in brackets, the experiment ID from the Genevestigator database are given. Expression ratios are colour coded, with bright yellow indicates a more than 8 fold higher expression in treated plants compared to control plants; bright blue indicates a more than 8 fold lower expression. Note that the data have been transposed relative to Figure 9 in order to fit the limitations of the spreadsheet. Click here for file

Additional file 5 **Oligonucleotide primers used for qRT-PCR**. Sequence information for oligonucleotide primers and probes used for RT-PCR of EREBP transcription factors. Click here for file
