# Supplementary material for: Comparative transcriptome analysis of Arabidopsis thaliana infested by diamond back moth (Plutella xylostella) larvae reveals signatures of stress response, secondary metabolism, and signalling
Source: BMC Genomics. 2008 Apr 9;9:154. doi: 10.1186/1471-2164-9-154 (PMC2375910; doi:10.1186/1471-2164-9-154)
Supplement: Additional file 5 — Oligonucleotide primers used for qRT-PCR. Sequence information for oligonucleotide primers and probes used for RT-PCR of EREBP transcription factors. [file 1471-2164-9-154-S5.pdf]

Additional File 5 - Primer pair and probe (reporter) set for real time RT-PCR of the transcription factors

AT1G28370	Forward:	GGTACTTTCGACACTCCTGAAGAAG
	Reverse:	TCCACGAAACTCAATAGCACGTT
	Probe:	CCGCTCGTGCCTACG
AT2G20880	Forwad:	ACAAAAGAGGACATCGTCGTTGA
	Reverse:	ACCCTGATTGATTTTCCCTTGTTCT
	Probe:	CTCCTCTTCTCTATGGCTACTG
AT4G34410	Forward:	CTCGGATTTAGTTTGACAGAAGAAGGA
	Reverse:	GACGAATTCGGTTCTGTTGTTAGG
	Probe:	ATAATGCATACCCGACTCTC
AT1G43160	Forward:	CCGGGTTACAACTTGTCTACCA
	Reverse:	CACCAGAGACAACATTAGTCAGCAT
	Probe:	ACACCATGTTTGAAATTG
AT5G13330	Forward:	CGCTCAGTTGCTTACGAGTAACAAT
	Reverse:	CGTTGAAAAAGGTTGACTGAAGAGA
	Probe:	ACGTGTAGTATGATAAATCA
AT3G57600	Forward:	CCAGCAAAGACTAGAAGAACTCAAGA
	Reverse:	GATTCGGTGGAGGAAGAACTAGAAG
	Probe:	AATAGGATTGAGATAAAAGTCC
AT1G24590	Forward:	CGTTTAGCTGACTTCCATGTCGAA
	Reverse:	TCAGCCCAACCTAACTCTCCAT
	Probe:	ATTCGGTCAGGTTAAATAT
